# Hydrogen Production by Steam Reforming of Ethanol over Nickel Catalysts Supported on Sol Gel Made Alumina: Influence of Calcination Temperature on Supports

**DOI:** 10.3390/ma6062229

**Published:** 2013-05-30

**Authors:** Zahira Yaakob, Ahmed Bshish, Ali Ebshish, Siti Masrinda Tasirin, Fatah H. Alhasan

**Affiliations:** 1 Department of Chemical and Process Engineering, Faculty of Engineering, Universiti Kebangsaan Malaysia (UKM), Bangi, Selangor 43600, Malaysia; E-Mails: aliebshish@gmail.com (A.E.); masrinda@eng.ukm.my (S.M.T.); 2 Catalysis Science and Technology Research Centre, Faculty of Science, Universiti Putra Malaysia, UPM Serdang, Selangor 43400, Malaysia; E-Mail: abuomohd999@yahoo.com

**Keywords:** ethanol reforming, hydrogen production, sol gel alumina, nickel catalyst

## Abstract

Selecting a proper support in the catalyst system plays an important role in hydrogen production via ethanol steam reforming. In this study, sol gel made alumina supports prepared for nickel (Ni) catalysts were calcined at different temperatures. A series of (Ni/Al_S.G._) catalysts were synthesized by an impregnation procedure. The influence of varying the calcination temperature of the sol gel made supports on catalyst activity was tested in ethanol reforming reaction. The characteristics of the sol gel alumina supports and Ni catalysts were affected by the calcination temperature of the supports. The structure of the sol gel made alumina supports was transformed in the order of γ → (γ + θ) → θ-alumina as the calcination temperature of the supports increased from 600 °C to 1000 °C. Both hydrogen yield and ethanol conversion presented a volcano-shaped behavior with maximum values of 4.3 mol/mol ethanol fed and 99.5%, respectively. The optimum values were exhibited over Ni/Al_S.G800_ (Ni catalyst supported on sol gel made alumina calcined at 800 °C). The high performance of the Ni/Al_S.G800_ catalyst may be attributed to the strong interaction of Ni species and sol gel made alumina which lead to high nickel dispersion and small particle size.

## 1. Introduction

Hydrogen is a potential source of clean energy mainly as fuel in fuel-cell systems, which are described as continuously operating batteries. It is also one of the cleanest and greenest sources of electrical energy [1,2]. The production of hydrogen by ethanol steam reforming was achieved using different catalytic systems. Our group [3] performed an extensive review on various catalytic systems employed in hydrogen production through ethanol reforming. Nickel (Ni) on different supports has been broadly investigated in ethanol steam reforming reaction because of its inexpensive nature and its wide use in the hydrogenation and steam reforming of hydrocarbons [4,5,6,7,8,9]. The activity of a catalytic system depends on several factors, such as active metal, nature of support, precursor used, method adopted for catalyst preparation, presence of promoters, and reaction conditions, including water-to-ethanol molar ratio, temperature, and space velocity. The nature of support has a significant effect on the characteristics and activity of catalysts. Conventional impregnation of commercial Al_2_O_3_ support is widely used in the synthesis of Ni-based catalysts because of its fast and simple operation [10,11,12,13,14,15,16]. Ni-based catalysts prepared using sol gel made γ-alumina support have higher hydrogen selectivity and ethanol conversion than those prepared using commercial γ-alumina support [17]. However, there are several reports about the use of nickel supported on sol gel made alumina for hydrogen production via steam reforming of natural gas [17,18,19]. A survey of literature showed that no work has been done so far on the production of hydrogen using nickel supported on sol gel made alumina catalyst via steam reforming of ethanol. The physical and chemical properties of alumina support are influenced by the treatment temperature of the alumina. Accordingly, the activity of Ni catalysts supported on sol gel made alumina (denoted as Al_S.G._) in ethanol steam reforming is influenced by the calcination temperature of the support. Thus, this study aims to prepare Al_S.G. _supports calcined at different temperatures. Ni/sol gel made alumina (denoted as Ni/Al_S.G._) catalysts were synthesized by an impregnation procedure and then tested for hydrogen production via ethanol steam reforming. The influence of the calcination temperature of Al_S.G._ on the activity of the Ni/Al_S.G._ catalysts in hydrogen production via ethanol reforming reaction, was studied.

## 2. Experimental Section

### 2.1. Catalyst Preparation

Al_S.G._ supports were prepared according to a previously described method [17]. Approximately 96 g of the precursor aluminum sec-butoxide (Sigma–Aldrich) was dissolved in 828 mL of ethyl alcohol under constant stirring at 80 °C (solution 1) to prepare 20 g of Al_2_O_3_ xerogel. HNO_3_ (1.37 mL) and distilled water (4.12 mL) diluted with 549 mL ethyl alcohol (solution 2) were mixed with solution 1. This mixture was fixed at 80 °C to form the sol. After cooling the sol, a transparent gel was obtained by slowly pouring 8.23 mL distilled water and 68.66 mL ethyl alcohol into the sol. The alumina gel was covered and kept for a day before it was dried overnight. The formed solid was calcined at different temperatures to obtain the alumina sol gel. This support was denoted as Al_S.G.T_, where T is the support calcination temperature (varied from 600 °C to 1000 °C at an interval of 100 °C).

Ni supported on sol gel alumina catalysts was synthesized by an impregnating amount of Ni(NO_3_)_2_·6H_2_O (Sigma–Aldrich) on the Al_S.G._ supports. The synthesized catalysts were denoted as Ni/Al_S.G.T_. The nickel content in all samples was fixed at 6 wt %.

### 2.2. Catalyst Characterization

The Brunauer–Emmett–Teller (BET) surface area, pore volume, and pore size distribution of the supports were measured by N_2_ adsorption at 77 K using a Micromeritics adsorption equipment (Model ASAP 2010, Micromeritics Instruments Inc., Norcross, USA) using N_2 _gas (99.99% purity).

The X-ray diffraction (XRD) patterns of the samples were recorded at 2θ ranging from 10° and 80° on a Bruker AXS D8 Advance diffractometer employing a scanning rate of 0.02° s^−1^ with Cu Kα radiation (λ = 1.5418). The working current and voltage of the X-ray tube were 30 mA and 40 kV.

Temperature-programmed reduction (TPR) measurements were performed for all catalysts using a Micromeritics instrument (Model Autochem II 2920, Quantachrome Corporation, FL, USA) connected to a thermal conductivity detector (TCD). The temperature was elevated from 25 °C to 900 °C at a rate of 10 °C min^−1^. For TPR analysis, a mixed stream of 5% H_2_–N_2_ mixture as carrier gas was passed on 0.03 g of catalyst sample at a flow rate of 20 cm^3^ min^−1^. Hydrogen chemisorption measurements were performed to determine the dispersion, particle size, and surface area of nickel. Initially, the system was purged with pure argon (30 mL min^−1^) from room temperature to 900 °C for 1 h. Prior to the chemisorption measurements, the catalyst was treated with a mixed stream of carrier nitrogen (20 mL min^−1^) and argon (20 mL min^−1^) by elevating the temperature from 25 °C up to 900 °C for 3 h to remove any impurities. After cooling down, the catalysts were reduced* in situ* with hydrogen at 400 °C for 1 h. The samples were then cooled to room temperature under argon flow (30 mL min^−1^). The quantity of hydrogen uptake was measured by passing diluted hydrogen (5.1% hydrogen in argon) through the catalyst. The dispersion, surface area, and mean particle size of nickel were evaluated according to Equations (1–3), respectively.

(1) $$D_{m}=\frac{V_{chem.}\times SF\times MW}{c/100}\;\times 100$$

(2) $$S.A._{m}=V_{chem.}\times 6.02\times 10^{23}\times SF\times \;\sigma_{m}\times 10^{-18}$$

(3) $$d=\frac{6\times c}{\left(S.A._{m}\right)\times \rho }\;\times 100$$

where,

$D_{m}$ = Metal dispersion
$V_{chem.}$ = Chemisorption volume (mol g^−1^)
SF = Stoichiometry factor = 1
MW = Supported metal atomic weight
c = Supported metal weight, wt %
$S.A._{m}$ = Metal surface area (per g catalyst)
$\sigma_{m}$ = Supported metal cross section area = 0.0649 nm atom^−1^
d = Mean particle diameter (nm)
ρ = Supported metal density

The amount of carbon deposited on the used catalyst surface was measured for each sample by performing CHNS elemental analysis with a Leco CHNS-932 Elemental Analyzer, and 2.073 mg of the used sample was treated at high temperature in air.

### 2.3. Catalyst Performance Test

Hydrogen production by ethanol steam reforming was used as a test reaction for all prepared catalysts. Reforming reaction was performed in a Pyrex glass tube reactor (internal diameter: 8 mm, length: 50 cm) at 400 °C and atmospheric pressure. Before the reaction takes place, the catalyst was heated up to 150 °C for 1 h to remove impurities and then reduced at reaction temperature* in situ* under flowing H_2_ for another 1 h. A mixture of liquid water and ethanol with a molar ratio 6:1 was introduced into the reactor containing 0.5 g of catalyst together with carrier N_2_ (30 mL min^−1^) gas. The reactant mixture was fed to the reactor by using a syringe pump (model NE-300) fixed at the required flow rate (0.1 mL min^−1^). The temperature inside the reactor was controlled and measured by using a thermocouple located in the catalyst bed. The catalytic data were recorded after 8 h reaction. The output gas stream was analyzed through gas chromatography (SRI 8610C Gas Chromatograph, USA) using a molecular sieve, a silica gel column, and a TCD with helium as the carrier gas. Each sample was analyzed at least twice, and the average was estimated. Condensed liquid was collected and analyzed by GC (SUPELCO) equipped with an Equity-1 capillary column (30 m × 0.32 mm × 0.1 μm) and a flame ionization detector.

The criteria used to evaluate catalyst performance included ethanol conversion and H_2_ yield. Equations (4) and (5) were used to calculate ethanol conversion and hydrogen yield, respectively.

(4) $$EtOH\;Conversion=\frac{F_{ETOH_{in}}-F_{ETOH_{out}}}{F_{ETOH_{in}}}x\;100$$

(5) $$H_{2}\;Yield=\frac{{F_{H_{2}}}_{,out}}{F_{ETOH_{in}}}$$

where $F_{ETOH_{in}}$ and $F_{ETOH_{out}}$ represent the molar flow rate at the ethanol inlet and outlet of the reactor, respectively, and ${F_{H_{2}}}_{,out}$ represents the flow rate at the hydrogen outlet of the reactor.

## 3. Results and Discussion

### 3.1. Support Physical Properties

**Figure 1:**
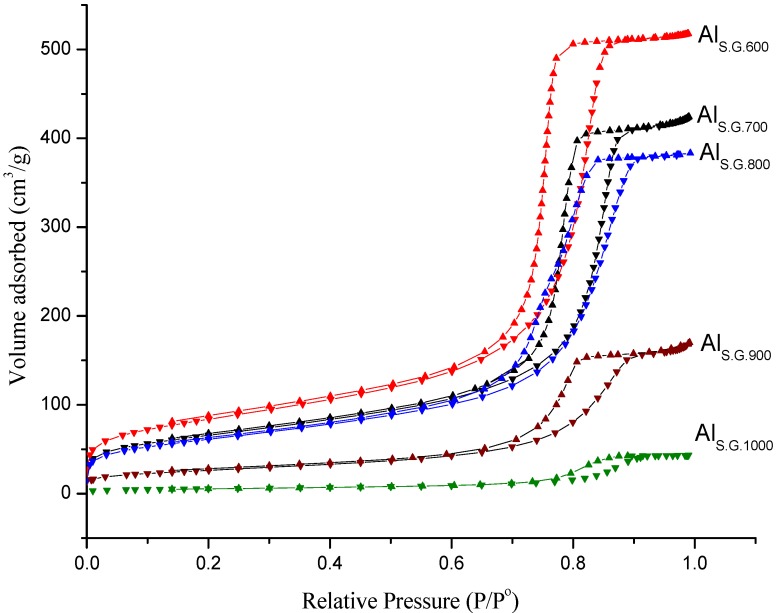
Nitrogen adsorption–desorption isotherms for sol gel alumina supports at different calcinations temperature.

The physical properties of Al_S.G. _calcined at different temperatures ranging from 600 °C to 1000 °C were tested by N_2_ adsorption–desorption isotherm determination (Figure 1). The samples exhibited type-IV (based on the International Union of Pure and Applied Chemistry) curves, indicating the presence of a mesoporous material. Interestingly, the calcination temperature of the supports showed an influence on hysteresis loops. That is, the hysteresis loops shifted to lower relative pressure with decreasing calcination temperature. This result indicates that the pore size of Al_S.G._ increased with increasing calcination temperature. This finding may be attributed to the development of pore texture with a relatively broad pore size distribution.

Table 1 summarizes the physical properties of the Al_S.G._ supports. The surface area of all prepared alumina supports decreased from 292 m^2^ g^−1 ^at 600 °C to 19 m^2^ g^−1 ^at 1000 °C. Similarly, pore volume decreased as the calcination temperature of the supports increased. Noticeably, the pore volume and surface area of the supports considerably declined at 1000 °C. By contrast, the pore diameter of Al_S.G._ increased with increasing calcination temperature. This result suggests that the physical properties of the Al_S.G._ supports were significantly influenced by calcination temperature which is agree with the data of previous studies [20,21,22].

**Table 1:** Physical properties of sol gel made alumina supports at different calcination temperatures.

Catalyst name	BET (m^2^/g) ^a^	Pore volume(cm^3^/g) ^b^	Pore diameter(nm) ^c^
Al_S.G.600_	292	0.81	7.91
Al_S.G.700_	230	0.67	8.46
Al_S.G.800_	214	0.60	9.06
Al_S.G.900_	92	0.27	9.10
Al_S.G.1000_	19	0.07	10.18

^a^ Calculated by the BET equation; ^b^ BJH desorption pore volume; ^c^ BJH desorption pore diameter.

### 3.2. Effect of Calcination Temperature on the Structure of the Supports and Supported Ni Catalysts

**Figure 2:**
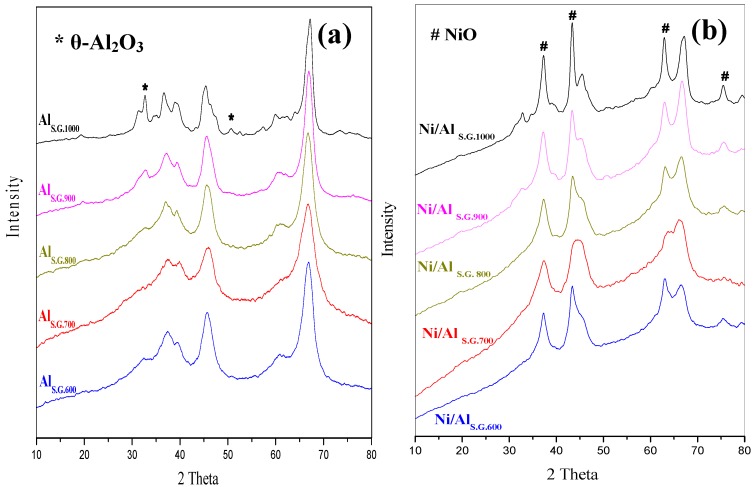
XRD patterns of (**a**) sol gel made alumina supports and (**b**) nickel catalysts supported on sol gel made alumina supports.

The effect of calcination temperature on the phase transformation and phases of the Al_S.G._ supports was tested by XRD measurements (Figure 2a). The supports calcined at temperatures ranging from 600 °C to 800 °C showed similar diffraction peaks belonging to γ-alumina. However, the alumina supports calcined at 900 °C exhibited two mixed phases of alumina (γ and θ) because the alumina support transformed from γ- to θ-alumina at this calcination temperature. As expected, the support calcined at 1000 °C showed characteristic peaks corresponding to θ-alumina. However, the α-alumina phase was not detected at this temperature. The transformation of alumina phases occurs because of dehydroxylation and sintering reactions [20,21], wherein the rate of these reactions varies by changing the calcination temperature of the support. Generally, the phase of γ-alumina can be detected at temperatures lower than or equal to 800 °C, whereas that of θ-alumina appears at temperatures above 800 °C and becomes more intense at 1000 °C.

The XRD diffraction peaks of the Ni/Al_S.G._ catalysts are shown in Figure 2b. All Ni/Al_S.G._ catalysts exhibited peaks belonging to nickel oxide (NiO). The diffraction peaks of NiO shifted to a higher angle as the calcination temperature of the support increased, as evidently observed in the Ni/Al_S.G1000_ catalyst. The strong and narrow NiO peaks exhibited over the Ni/Al_S.G1000_ catalyst can be ascribed to the weak interaction between Ni species and the Al_S.G._ supports. By contrast, the high surface area of the supports over the catalysts (Ni/Al_S.G600_, Ni/Al_S.G700_, and Ni/Al_S.G800_) enhanced the incorporation of alumina cation into the NiO lattice.

### 3.3. Metal–Support Interaction

**Figure 3:**
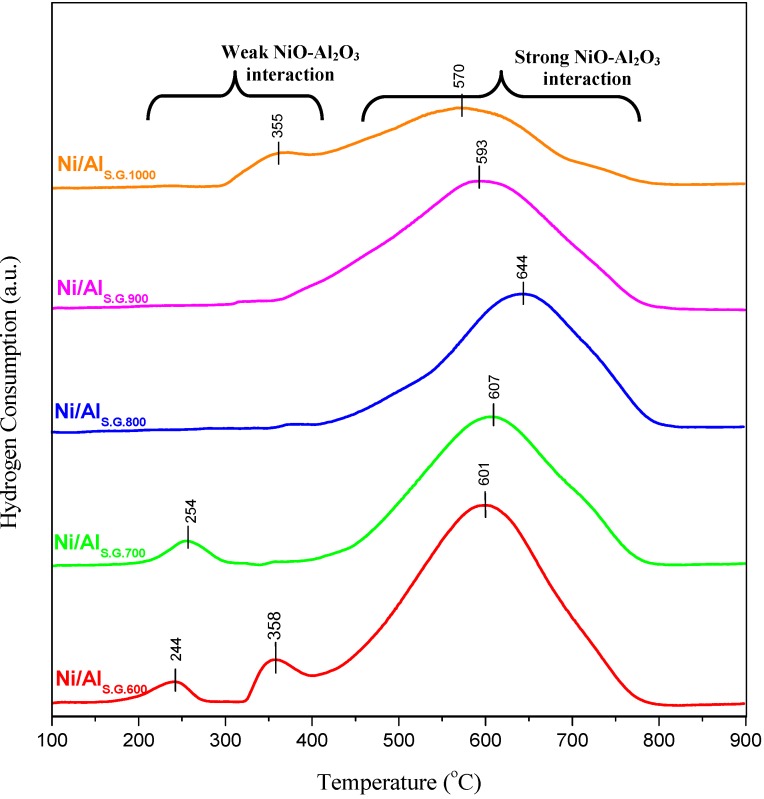
Temperature-programmed reduction (TPR) profiles of nickel/sol gel alumina catalysts.

The TPR profiles of the Ni/Al_S.G._ catalysts are shown in Figure 3 to study the metal–support interaction. Two zones of reduction peaks are presented. The first zone was in the range of 200 °C to 370 °C, which was observed in the Ni/Al_S.G600_, Ni/Al_S.G700, _and Ni/Al_S.G1000 _samples. These reduction peaks correspond to the reduction of the NiO species which has minimal or no interaction with the Al_2_O_3_ supports [23,24]. The other zone of reduction peaks was observed in all samples and located in the range of 450 °C to about 800 °C, which may be attributed to the strong interaction of NiO and alumina support. As reported elsewhere, the formation of strongly interacting NiO-Al_2_O_3_ phase was observed in the region between 550 °C and 750 °C [25,26]. Notably, as the calcination temperature of the support increased (Ni/Al_S.G600_, Ni/Al_S.G700_, and Ni/Al_S.G800_), the reduction bands shifted to higher temperature values until they reached a maximum value of 644 °C over the Ni/Al_S.G800_ catalyst. As a result, more NiO species were embedded more deeply in the Al_2_O_3_ lattice, thereby strengthening the interaction between the metal oxide and the carrier. This strong interaction promotes the distribution of the NiO species on the catalyst, which will further affect the steam reforming reaction. A further increase in the calcination temperature of the support beyond 800 °C (Ni/Al_S.G900_ and Ni/Al_S.G1000_) caused reduction bands to start to shift to lower temperature values. Consequently, more NiO species were located on the support surface, which indicates weaker interaction between the metal and the carrier as it caused nickel agglomeration and further affected the activity of the catalyst. Interestingly, bands belonging to Ni aluminate were not observed on all the examined catalysts, which coincides well with the XRD results.

### 3.4. Hydrogen Chemisorption Measurements

The amount of H_2_ uptake, and dispersion, surface area, and mean particle size of nickel were determined through chemisorption measurements, as shown in Table 2. As a result of hydrogen chemisorption, the amount of hydrogen uptake over the Ni/Al_S.G600_, Ni/Al_S.G700_, and Ni/Al_S.G800_ catalysts increased as the calcination temperature increased. Furthermore, an increase in calcination temperature (Ni/Al_S.G900_, Ni/Al_S.G1000_) led to a decrease in hydrogen uptake. The trend in the dispersion and surface area of nickel is proportional to the amount of hydrogen uptake. As a result, the dispersion and surface area of nickel increased in the following order: Ni/Al_S.G600_ < Ni/Al_S.G700_ < Ni/Al_S.G1000_ < Ni/Al_S.G900_ < Ni/Al_S.G800_, whereas the mean particle size values of nickel decreased. These results are also supported by TPR experiments, as shown in Figure 3.

**Table 2:** Results for hydrogen chemisorptions measurements.

Catalyst name	Amount of H_2_ uptake (μmol g^−1^)	Nickel dispersion (%) *	Nickel surface area (m^2^ g^−1^) *	Mean particle diameter (nm)
Ni/Al_S.G.600_	45	4.40	1.75	22.9
Ni/Al_S.G.700_	254	24.8	9.90	4.1
Ni/Al_S.G.800_	566	55.4	22.1	1.8
Ni/Al_S.G.900_	489	47.8	19.1	2.1
Ni/Al_S.G.1000_	310	30.3	12.1	3.3

* H/Ni_atom_ = 1 was assumed.

### 3.5. Steam Reforming of Ethanol over the Supported Ni Catalysts

**Figure 4:**
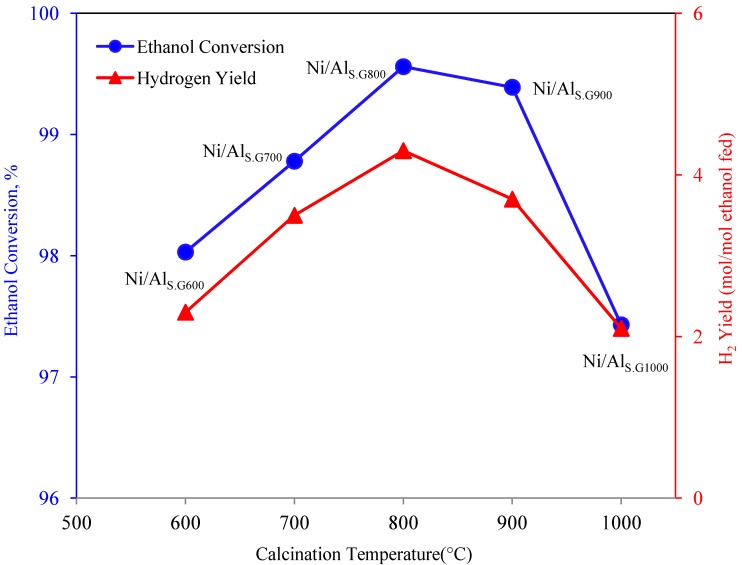
Ethanol conversion and hydrogen yield over Ni/sol gel made alumina catalysts in the steam reforming of ethanol, plotted as a function of calcinations temperature of the support. The catalytic data were obtained after 8 h reaction.

The ethanol conversion and hydrogen yield over the Ni/Al_S.G. _catalysts in the ethanol reforming reaction as a function of the calcination temperature of Al_S.G._ are shown in Figure 4. Both ethanol conversion and hydrogen yield were significantly affected by the calcination temperature of the Al_S.G._ supports. Catalyst deactivation was tested by evaluating the deposited carbon on the used samples to compare the performance of each catalyst after 8 h reaction. CHNS elemental analyses were performed to detect the quantity of carbon deposited on the used samples, as shown in Table 3. All samples contain only trace amounts of carbon. Therefore, catalyst deactivation by carbon deposition can be inferred to have an insignificant effect on catalytic activity after an 8 h run. The increase in both ethanol conversion and hydrogen yield was in the following order: Ni/Al_S.G1000 _< Ni/Al_S.G600 _< Ni/Al_S.G700_ < Ni/Al_S.G900_ < Ni/Al_S.G800_. Among all the evaluated catalysts, the Ni/Al_S.G800 _catalyst exhibited the best catalytic performance. The high performance of the Ni/Al_S.G800 _catalyst may be due to its physical and chemical characteristics. Although the surface area and pore volume of the supports of the Ni/Al_S.G600 _and Ni/Al_S.G700 _catalysts were higher than those of the Ni/Al_S.G800 _catalyst (Table 1), the Ni species in the Ni/Al_S.G800 _catalyst interacted strongly with the alumina support (Figure 3). This strong metal–support interaction over the Ni/Al_S.G800 _catalyst not only improves the dispersion and surface area of nickel but also prevents metal agglomeration (Table 2). As a result, the number of active sites that can facilitate a reaction increased because of high metal dispersion. Consequently, this will enhance catalytic behaviour during the steam reforming reaction. Moreover, high dispersion of metal particles on the Ni/Al_S.G800 _catalyst could exhibit high resistance toward carbon formation. An attempt has been done by Seo* et al.* [19] to correlate the catalytic activity and selectivity of Ni/Al_S.G._ catalyst for steam reforming of natural gas and concluded that the support calcined at 900 °C is the most active system for hydrogen production, while our catalyst system shown highest selectivity and activity at calcined temperature of 800 °C.

**Table 3:** Carbon deposited on the used catalysts after 8 h reaction.

Catalyst name	Amount of carbon deposition (wt %)
Ni/Al_S.G.600_	0.26
Ni/Al_S.G.700_	0.20
Ni/Al_S.G.800_	0.14
Ni/Al_S.G.900_	0.11
Ni/Al_S.G.1000_	0.68

## 4. Conclusions

Al_S.G._ supports were prepared and calcined at different temperatures. Ni/Al_S.G._ catalysts were synthesised by an impregnation method and then tested for H_2_ production via ethanol steam reforming. The influence of the calcination temperature of the Al_S.G._ supports on the activity of the Ni/Al_S.G._ catalysts was studied. The structure of Al_S.G._ was converted from γ-alumina (from 600 °C to 800 °C) to a mixed phase between γ- and θ-alumina (at 900 °C) and then to θ-alumina (at 1000 °C). The increase in both ethanol conversion and hydrogen yield was in the following order: Ni/Al_S.G800_ > Ni/Al_S.G900_ > Ni/Al_S.G700_ > Ni/Al_S.G600_ > Ni/Al_S.G1000_. Ni/Al_S.G800 _exhibited the best catalyst activity in terms of hydrogen yield and ethanol conversion. The strong metal–support interaction of the Ni/Al_S.G800_ catalyst enhanced the dispersion and surface area of nickel while reducing mean particle size values, thereby resulting in high catalytic performance in terms of hydrogen yield.
